# The Phylodynamics of Seasonal Influenza A/H1N1pdm Virus in China Between 2009 and 2019

**DOI:** 10.3389/fmicb.2020.00735

**Published:** 2020-04-28

**Authors:** Yingying Ma, Kai Liu, Yong Yin, Jianru Qin, Yan-Heng Zhou, Juan Yang, Shenwei Li, Leo L. M. Poon, Chiyu Zhang

**Affiliations:** ^1^Chinese Academy of Sciences (CAS) Key Laboratory of Molecular Virology & Immunology, Institut Pasteur of Shanghai, Chinese Academy of Sciences: University of Chinese Academy of Sciences, Shanghai, China; ^2^Department of Pulmonary, Shanghai Children’s Medical Center Affiliated to Shanghai Jiao Tong University School of Medicine, Shanghai, China; ^3^College of Life Sciences, Henan Normal University, Xinxiang, China; ^4^Key Laboratory of Public Health Safety, Ministry of Education, Fudan University School of Public Health, Shanghai, China; ^5^Shanghai International Travel Healthcare Center, Shanghai, China; ^6^School of Public Health, The University of Hong Kong, Hong Kong, China

**Keywords:** A/H1N1pdm, genetic drift, genetic diversity, transmission, viral evolution

## Abstract

Since its first introduction into China in 2009, influenza A/H1N1pdm virus has undergone a rapid expansion and replaced the classical seasonal A(H1N1) virus. To characterize the ongoing evolution and national transmission dynamics of this virus, we analyzed 335 complete genome, 1259 HA, and 1043 NA sequences of the A/H1N1pdm strains detected in China. We found that the dN/dS value and relative genetic diversity of the A/H1N1pdm virus experienced a decrease from 2009 to 2017, and then a rapid increase during 2018–2019. Importantly, elevated relative genetic diversity was observed in the A/H1N1pdm and the A/H3N2 viruses, as well as two lineages (Victoria and Yamagata) of influenza B virus during 2018–2019, suggesting the simultaneous changes of these viruses in terms of genetic diversity might be associated with the recent large outbreak of seasonal influenza epidemic in China during 2018–2019. Fifteen amino acid mutations were found to be fixed along the main trunks of both HA and NA phylogenetic trees, and some of them are located in the antigen binding site or the receptor binding site. A sequential accumulation of mutations relative to the 2009-vaccine strain was observed in the circulating A/H1N1pdm strains during 2009–2016, while a rapid accumulation of mutations relative to the 2015-vaccine strain appeared in the emerging variants in 2017 shortly after the release of the vaccine. Multiple introductions of the A/H1N1pdm lineages into China were observed during 2009–2019, and East China and South China were found to serve as two major epicenters responsible for the national migration of the virus. In summary, these data provide important insights into the understanding of the evolution, epidemiology and transmission of the A/H1N1pdm virus, and highlight the importance of strengthening influenza surveillance in East China and South China.

## Introduction

Seasonal influenza epidemics are a major public health burden, causing significant morbidity and mortality all over the world (Nelson and Holmes, 2007). Currently, seasonal influenza-associated deaths were estimated to be 291,000–645,000 per year (Iuliano et al., 2018). Two subtypes of influenza A virus (A/H1N1 and A/H3N2), and two lineages of influenza B virus (Victoria and Yamagata) are major causative agents responsible for the seasonal influenza epidemics, and often co-circulating. The classical seasonal A(H1N1) had circulated in humans from 1918 to 1957 and from 1977 to 2009 (Snacken et al., 1999). However, a novel swine-origin H1N1 influenza virus (A/H1N1pdm) emerged in humans during March-April 2009 in Mexico (Smith et al., 2009; Vijaykrishna et al., 2010). It spread rapidly to 168 countries/regions around the world, and led to a global pandemic with an estimated death of > 123,000 by the end of 2009 (Simonsen et al., 2013). Since 2009, the A/H1N1pdm completely replaced the classical seasonal A(H1N1) virus and exhibited similar seasonal epidemic characteristics thereafter.

Although influenza virus vaccines are effective in preventing the spread of seasonal influenza virus, error-prone trait of viral RNA polymerase enables the virus to generate new variants to escape the immunity induced by vaccination or prior infections (Sandbulte et al., 2011; Han et al., 2019). The evolutionary and epidemiological dynamics of A/H3N2 and two lineages of influenza B virus have been well characterized at the global scale (Bedford et al., 2015; Vijaykrishna et al., 2015; Langat et al., 2017; Geoghegan et al., 2018). In 2015, a study characterized the early phylodynamics of the A/H1N1pdm virus from 2009 to 2014, and demonstrated that the A/H1N1pdm virus experienced an evolution shift from early adaptions to the new human host to subsequent escape from pre-existing host immunity (Andrews et al., 2015; Debbink et al., 2017), which is accompanied with a reduced antigenic/genetic diversity (Su et al., 2015). Under host immune pressure and periodic bottleneck, A/H1N1pdm virus evolved to a seasonal epidemic pattern (Varble et al., 2014; Su et al., 2015).

The A/H1N1pdm virus was introduced in China in 2009, and caused 793 deaths by the February 2010 (Yu et al., 2011). In response to the pandemic of A/H1N1pdm, routine monitoring of A/H1N1pdm virus was promptly included into a national surveillance system for influenza in China since 2009. However, phylodynamics of the A/H1N1pdm in China is poorly understood. Furthermore, the majority of previous evolutionary analyses of influenza viruses were conducted before 2015, which leaves a big gap on the ongoing evolution of the A/H1N1pdm virus from 2014 to nowadays.

Since the winter of 2017, higher level of seasonal influenza activity occurred in China and other countries (Gao, 2018; Barr et al., 2019; Fu et al., 2019). For example, about 63.3% (405/640) children with influenza were associated with influenza A viruses in Shanghai (unpublished data). The evolutionary reason for the 2017–2019 large outbreak of seasonal influenza remains to be answered. To well understand the evolutionary and spatiotemporal dynamics of the A/H1N1pdm virus in China from 2009 to 2019 and to investigate the evolutionary reason for the 2017–2019 large outbreaks, we performed in-depth phylogenetic analyses of all publicly available sequence data of the virus from China together with genome sequences generated from Shanghai in this study. Our results provide new insights to the spatiotemporal evolution and migration dynamics of the A/H1N1pdm virus in China from 2009 to 2019.

## Methods

### Sample Collection, and Amplification and Sequencing of Whole Genome of Influenza A From Shanghai

Nasopharyngeal swabs from children with symptoms of influenza-like illness (ILI) were collected from Shanghai Children’s Medical Center from 2017 to 2019. The collection of samples was approved by the Ethics Committees of Shanghai Children’s Medical Center. Oral or written informed consents were obtained from children’s parents or guardians before sample collection. Infection with influenza A was determined by Colloidal gold immunochromatographic assay (Wondfo Co., Ltd, China) and an in-house RT-qPCR assay specific for influenza A virus. From the influenza A virus positive samples by both assays, we randomly selected 99 for further amplification and sequencing of complete genome.

RNA was extracted by Trizol LS reagent (Invitrogen, United States). cDNA was first generated by SuperScript^®^ III First-Strand Synthesis System for RT-PCR (Invitrogen, United States). Universal primers MBTuni12 and MBTnui13 were used to amplify all 8 influenza A viruses genomic segments as described previously (Zhou et al., 2009). Two additional reactions for PB1 and PB2 genomic segments were separately performed using primer sets of Ba-PB2-1 and Ba-PB2-2341R, and Bm-PB1-1, and Bm-PB1-2341R (Hoffmann et al., 2001). The PCR products were verified by gel electrophoresis, and purified using a QIAquick PCR Purification kit (Qiagen, Germany). Three purified DNA products were pooled together and fragmented with Covaris system (Covaris Inc., United States) for ∼200 bp. DNA libraries with unique indexes were constructed using Hieff NGSTM MaxUpII DNA Library Prep Kit for Illumina^®^ according to manufacturer’s instructions, and mixed in equal concentrations for next generation sequencing (NGS). NGS was performed on Illumina HiSeq X Ten platform by Novogene Co., Ltd. The sequencing depth was about 0.3G for each sample (about 20,000 × genome coverage). The raw data were deposited in the Sequence Read Archive (SRA) database (study PRJNA609449).

### Sequencing Data Assembly and Annotation

The raw data of NGS was first cleaned using Cutadapt v1.18 by removing Illumina sequencing adaptor. The low-quality sequences were removed under the parameters of SLIDINGWINDOW: 4:20 and MINLEN:50 using Trimmomatic v0.38 (Bolger et al., 2014). Host contaminated reads were filtered out by Bowtie2 v2.3.4.3 (Langmead and Salzberg, 2012). The remaining high-quality reads were *de novo* assembled by Megahit (v1.1.3) (Li et al., 2015). Contigs were de-replicated, putative chimeric sequences were removed, and the remaining contigs were clustered at 98% identity using Vsearch v2.10.4 (Rognes et al., 2016). Contigs with a length of more than 500nt were further mapped to the INFLUENZA database (download on October 25, 2018) by Blastn (version 2.7.1+) (Camacho et al., 2009) and unique genomic sequences containing the complete ORFs of each influenza genomic segment were generated. Only the assembled genomic sequence with more than 80% genomic coverage were used. A total of 99 genomic sequences of influenza A were obtained, and deposited in GISAID database^1^ (Supplementary File S1). A consensus genomic sequence of each sample was used in further phylogenetic analysis.

### Sequence Data of the A/H1N1pdm From China

We downloaded all available HA and NA sequences of the A/H1N1pdm strains isolated in China from NCBI Influenza Virus Resource and GISAID EpiFlu database by 12 June, 2019. After discarding low-quality sequences with gap or incomplete coding region, and 100% identical sequences, 1,259 HA and 1,043 NA sequences of the A/H1N1pdm were obtained. Furthermore, all available full-genome sequences of the A/H1N1pdm from China were also downloaded to the database by 12 June, 2019, and a total of 320 full genomes were obtained.

### Phylogenetic Analysis

The HA and NA sequences were separately aligned by MAFFT v7.425 (Katoh et al., 2002), and subjected to the construction of maximum likelihood (ML) trees. To investigate the evolutionary history of A/H1N1pdm in China, the phylogenetic trees of HA and NA genes were constructed using the maximum likelihood (ML) method in PhyML3.0, with the general time reversible (GTR) model of nucleotide substitution selected in jModelTest2.1.10. The reliability of the phylogenetic trees was evaluated with 1000 replications. Ancestral codon substitutions at each major tree node was inferred using MEGA7.0. To visualize the locations of the amino acid substitutions involving in receptor binding and genetic drift, these sites were mapped onto a three-dimensional structure of the H1-HA of the A/H1N1pdm (Protein Data Bank code: 3LZG) using PyMol2.3.0. The divergence time was assessed by plotting root-to-tip divergence versus year of isolation using the resulting phylogenetic trees with TempEst v1.5.1 (Rambaut et al., 2016).

### Natural Selection

The ratio of non-synonymous to synonymous substitution rates (dN/dS) was used to test whether the A/H1N1pdm was under positive selection. The dN/dS ratio was an average over all sites and lineages that was estimated using the M0 model of Codeml program implemented in PAML4.8 (Yang, 2007). To access the adaptive evolution process of A/H1N1pdm from 2009 to 2019, we divided the A/H1N1pdm sequences into six periods of 2009–2010, 2011–2012, 2013–2014, 2015–2016, 2016–2017, and 2018–2019, and calculated the dN/dS ratio of each time period. Furthermore, the positively selected sites for each gene was inferred using the MEME method in Datamonkey^2^ and the Bayesian Experience Bayesian (M8 model + BEB) method in PAML4.8. To see whether reassortment occurs in evolution of the A/H1N1pdm virus, tanglegrams were generated based on the ML phylogenies of eight genomic segments using the software Dendroscope v.3.6.3 (Huson and Scornavacca, 2012).

### Temporal Dynamics of the A/H1N1pdm

To estimate the evolutionary rate and the time to the most recent common ancestor (tMRCA) of the A/H1N1pdm in China, about 370 sequences of each genomic segment were subjected to Bayesian molecular clock analyses using the Markov chain Monte Carlo (MCMC) method implemented in BEAST v.1.10.4 (Suchard et al., 2018). Marginal likelihood estimation (MLE) and Bayes factors (BF) were used to select the best coalescent prior and molecular clock model for the data (Brynildsrud et al., 2018). A strict molecular clock with a Hasegawa-Kishino-Yano (HKY) nucleotide substitution model with a gamma distributed model of among site rate variation using four rate categories was used. At least two independent MCMC analyses were run for each segment of the A/H1N1pdm for 300 million generations sampled every 30,000 runs. The genome-wide relative genetic diversity of each genomic segment was estimated using Gaussian Markov random field (GMRF) Bayesian Skyride. The BEAGLE library was used to improve computational performance. Parameter convergence was assessed using Tracer v.1.7.1, and a minimum effective sample size (ESS) of 200 was required. After the removal of 10% burn-in, a summary Maximum Clade Credibility (MCC) tree was inferred and viewed using Figtree v1.4.4.

To understand the evolutionary dynamics of seasonal influenza in China from 2009 to 2019, all available 1,192 HA sequences of the A/H1N1pdm from China were further analyzed, and compared with A/H3N2, influenza B/Victoria and influenza B/Yamagata. There are 1,021, 545, and 538 HA sequences for A/H3N2, influenza B/Victoria and influenza B/Yamagata from China to be downloaded from GenBank and GISAID, respectively. A strict molecular clock, the SRD06 codon position model, HKY85 plus gamma substitution model, and a GMRF Bayesian Skyride in BEAST v.1.10.4 were used to infer the phylogeny and the population dynamics of the seasonal influenza viruses.

### Discrete Phylogeography Analyses

To clarify the spatial dynamics of the A/H1N1pdm in China, Bayesian phylogeography analysis was carried out to reconstruct the ancestral geographical area, and to infer the migration patterns using BEAST v.1.10.4. Each HA sequence was assigned two characters reflecting its sampling time and geographic location, and the geographic location was defined as one of the seven major regions: North (Beijing, Hebei, Inner Mongolia, Shanxi, and Tianjin), East (Anhui, Fujian, Jiangsu, Jiangxi, Shanghai, Shandong, and Zhejiang), Central (Henan, Hubei, and Hunan), Northwest (Gansu, Qinghai, Ningxia, Shaanxi, and Xinjiang), South (Guangdong, Guangxi, and Hainan), Southwest (Chongqing, Guizhou, Sichuan, Tibet, and Yunnan), and Northeast China (Heilongjiang, Jilin, and Liaoning). An asymmetric substitution model with stochastic search variable selection, a strict clock model and exponential growth coalescent prior were used to estimate the diffusion rates among locations. Two independent analyses were run using 500 million generations, and sampling was performed every 50,000 generations. The BF was used to determine the statistical significance for the diffusion between discrete locations. The support level of the diffusion route is defined as follows: BF ≥ 1000, 100 ≤ BF < 1000, 10 ≤ BF < 100, and 3 ≤ BF < 10 indicate decisive, very strong, strong and substantial support, respectively.

## Results

We obtained 99 seasonal influenza A virus genome sequences, including 66 A/H1N1 and 33 A/H3N2, from children in Shanghai during 2017–2019 using NGS. The deduced sequences have been deposited in GISAID database (Supplementary File S1). All A/H1N1 strains belong to the A/H1N1pdm, including 36 from 2018, and 30 from 2019. The A/H3N2 strains include 13 and 20 from 2018 and 2019, respectively.

### Genome Evolution of the A/H1N1pdm in China

We constructed MCC trees using eight influenza genomic segments to understand the evolution of the A/H1N1pdm in China. The A/H1N1pdm showed an explosive epidemic during 2009–2010, and then exhibited a ladder-like onward phylogeny since 2011 (Supplementary Figure S1). The A/H1N1pdm strains detected within the same season generally formed a specific lineage. The existing lineage in one season was often replaced by a newly emerging lineage in the following year except that few lineages appeared to extend to the following year possible due to the same winter-spring epidemic season (Supplementary Figure S1). Interestingly, the strains from 2019 were dispersed within the strains from 2018, and did not form independent clade, which is much like the characteristic of the strains during 2009–2010. The regression analysis of root-to-tip genetic distance against sampling time revealed a strong clock-like onward evolution of the A/H1N1pdm virus with a well linear accumulation of nucleotide divergence over time (*R*^2^ = 0.98) (Supplementary Figure S2). The evolutionary rate of the A/H1N1pdm was estimated to be between 2.8–4.4 × 10^–3^ substitutions per site per year for eight genomic segments, with the highest substitution rate in HA gene (95% highest posterior density (HPD): 3.8–4.9 × 10^–3^) and the lowest rate in PB1 gene (95% HPD: 2.4–3.1 × 10^–3^) (Table 1). The origin time of the A/H1N1pdm virus was estimated to be during 2008.6–2009.2, which was very consistent with the previous estimate (Table 1; Su et al., 2015).

**TABLE 1:** Estimated time of most recent common ancestor (TMRCA) and nucleotide substitution rate of A/H1N1pdm virus in China from 2009 to 2019.

**Gene**	**TMRCA of root height (mean)**	**95% HPD lower**	**95% HPD upper**	**Mean substitution rate**	**95% HPD lower**	**95% HPD upper**
PB2	2009.0	2008.8	2009.2	3.0E−03	2.6E−03	3.4E−03
PB1	2008.9	2008.6	2009.1	2.8E−03	2.4E−03	3.1E−03
PA	2008.6	2008.1	2009.0	3.0E−03	2.6E−03	3.4E−03
HA	2009.0	2008.8	2009.1	4.4E−03	3.8E−03	4.9E−03
NP	2009.0	2008.8	2009.2	3.3E−03	2.8E−03	3.8E−03
NA	2009.0	2008.7	2009.2	3.8E−03	3.3E−03	4.4E−03
MP	2009.2	2009.0	2009.3	3.8E−03	3.1E−03	4.6E−03
NS	2009.0	2008.7	2009.3	4.2E−03	3.3E−03	5.1E−03

The reassortment events during the evolution of the A/H1N1pdm virus were assessed by comparing topological congruence between eight genomic segments (Supplementary Figures S3–S9). The HA tanglegram was rather incongruent with those of other genomic segments, indicating that the intra-subtype reassortments were frequent during the evolution of the A/H1N1pdm virus. There are two periods (2009–2010 and 2018–2019) that the virus experienced very frequent reassortments. During 2009–2010, the reassortments were observed between HA and other seven genomic segments, while during 2018–2019, the reassortments occurred more frequently between HA and internal genomic segments, especially PB2, NP, MP, and NS.

**FIGURE 1:**
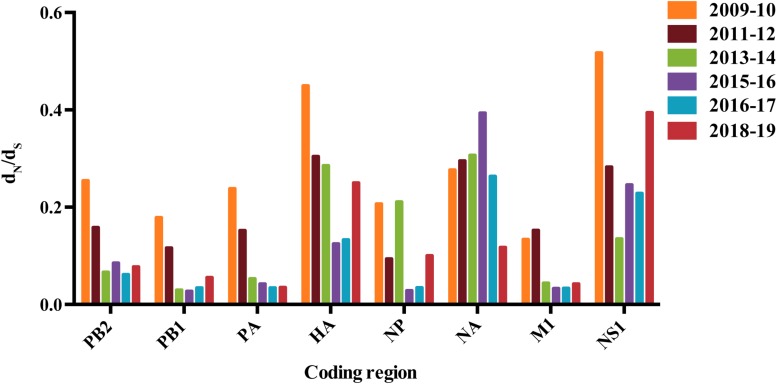
The dN/dS values for major genes of A/H1N1pdm virus. Colored bars correspond to different years. This dN/dS value represents an average over all sites and lineages.

Analysis of the ratio of the number of non-synonymous to synonymous substitutions per site (dN/dS) revealed significant difference among eight gene segments of the A/H1N1pdm virus (Figure 1). Higher dN/dS ratios were observed in HA, NA, and NS genes, implying stronger selection pressure acting on these genes. Interestingly, we found a decreasing trend of the dN/dS ratio in almost all gene segments except NA that was converse from 2009 to 2017 (Figure 1). Elevated dN/dS ratio occurred in HA, NS1, and NP genes during the 2018–2019 pandemic period, while the opposite was observed in NA gene. The changes in dN/dS ratio over time appeared not to be affected by the sampling bias (i.e., greater sampling density) in some years (Supplementary Figure S10).

### Population Genetics of the A/H1N1pdm in China

**FIGURE 2:**
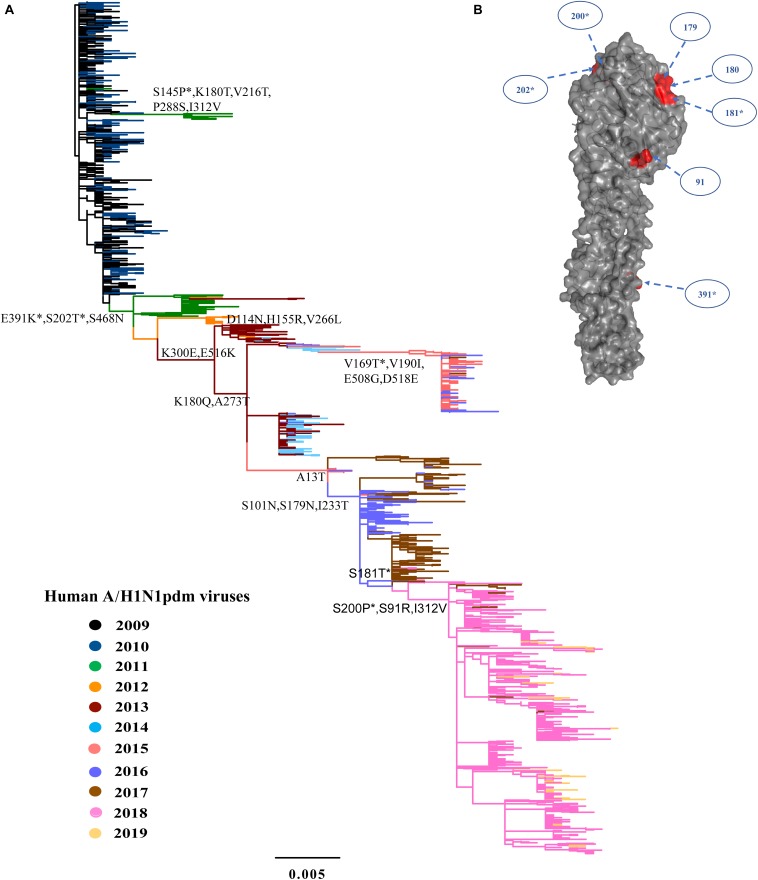
Phylogeny of A/H1N1pdm virus by HA gene and three-dimensional structure of HA glycoprotein. **(A)** Maximum likelihood tree inferred from 1259 A/H1N1pdm virus HA sequences in China from 2009 to 2019. The color of the branch is marked by the isolation year of the strain. Fixed amino acid mutations are mapped at the major nodes of the tree. Positively selected sites are highlighted by asterisks (*). Scale bar represents number of substitutions per site. **(B)** Three-dimensional structure map of HA monomer of A/H1N1pdm virus (Protein Data Bank code: 3LZG). Antigenic sites were shown in red in the three-dimensional structure.

**FIGURE 3:**
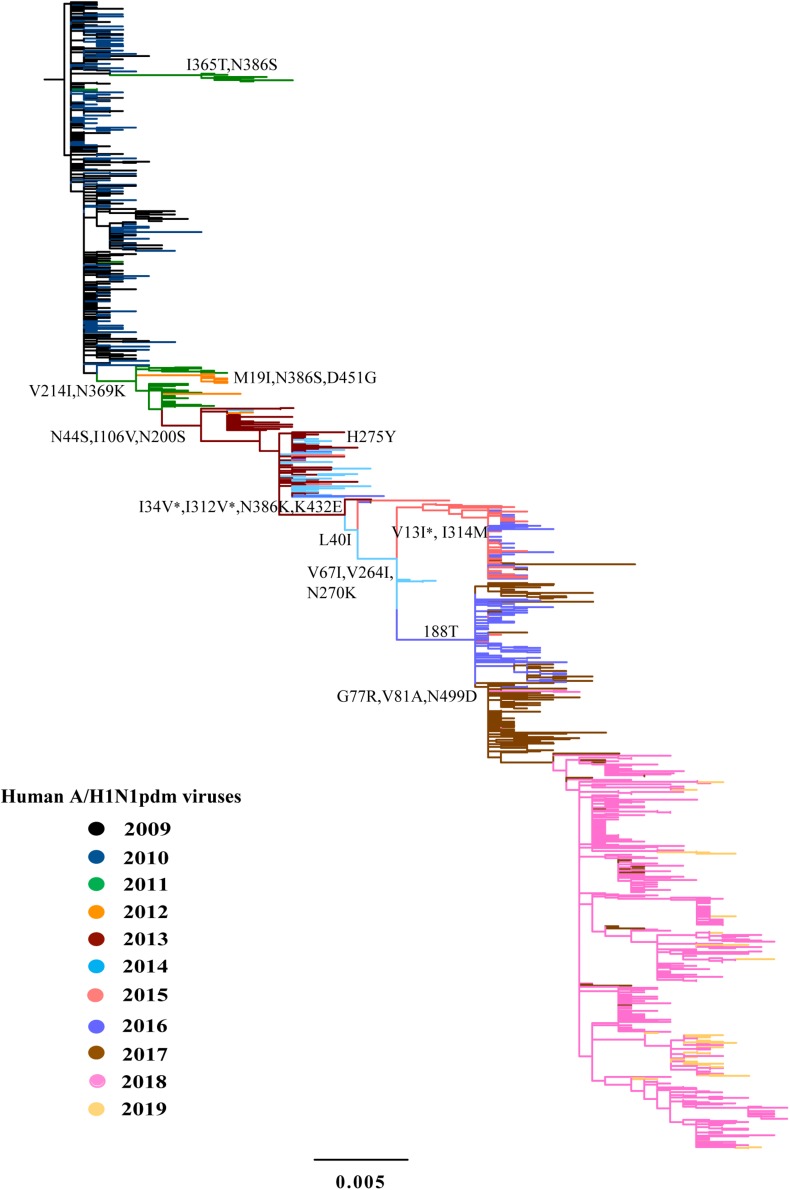
Phylogeny of A/H1N1pdm virus by NA gene. The ML tree inferred from 1043 A/H1N1pdm virus NA sequences in China from 2009 to 2019. The color of the branch is marked by the isolation year of the strain. Fixed amino acid mutations are mapped at the major nodes of the tree. Positively selected sites are highlighted by asterisks (*).

The analyses of all available HA and NA sequences from China showed similar topologies of phylogeny to the complete genomic sequences (Figures 2A, 3 and Supplementary Figure S1). Because each year covers two epidemic seasons, the spring of one season and the winter of next season, multiple lineages of the A/H1N1pdm virus appeared to co-circulate in China in each year. The existing lineages in one winter-spring epidemic season were replaced by the subsequently emerging lineages.

We inferred the ancestral sequences at each node of the HA ML tree. Fifteen amino-acid substitutions were fixed along the backbone of the HA phylogeny, and the majority of these amino-acid substitutions occurred after 2011 (Figure 2A). Among these fixed amino-acid mutations, four were also identified as positively selected sites by both the M8 model in PAML4.8 and the mixed effects model of evolution (MEME) method in DataMonkey (Supplementary Table S1). In particular, majority of the fixed mutations are located in HA1 subunit, and only a few were located in the stem area (Figure 2B). Six sites were located either at antigenic sites or in proximity to the receptor-binding site (RBS), including S91R (Cb antigenic site), S202T (Sb antigenic site), S179N-180Q-181T (Sa antigenic site), and S200P [antigenic epitope (190-helox) adjacent to RBS]. Half of the six fixed sites occurred after 2017 (Figure 2 and Table 2). For NA gene, there were fifteen amino acid substitutions to be fixed along the backbone of the phylogeny. Two amino acid substitutions (N369K and I314M) were involved in antigenic sites (Figure 3 and Table 2). Among these fixed amino-acid mutations, three were also identified as positively selected sites by the M8 model in PAML4.8 (Figure 3 and Supplementary Table S2), and no positively selected sites were identified in antigenic sites of NA gene (Table 2). Distinct from the HA gene, most amino acid mutations in NA gene were fixed during 2013–2017 (Table 2).

**TABLE 2:** Amino acid variations along the major trunk of the phylogenetic trees of A/H1N1pdm virus HA and NA glycoproteins over time.

**Gene**	**Amino acid position**	**2010**	**2011**	**2012**	**2013**	**2014**	**2015**	**2016**	**2017**	**2018**	**Structural mapping (solvent exposure and antigenic epitope location)**
HA	391	E391K									
	202		S202T								Sb antigenic sites
	468		S468N								
	114			D114N							
	180				K180Q						Sa antigenic sites
	273				A273T						
	300				K300E						
	516				E516K						
	101							S101N			
	179							S179N			Sa antigenic sites
	233							I233T			
	181								S181T		Sa antigenic sites
	91									S91R	Cb antigenic sites
	200									S200P	Antigenic epitope (190-helix), adjacent to RBS
	312									I312V	
NA	241		V241I								
	369		N369K								Antigenic sites
	44				N44S						
	106				I106V						
	200				N200S						
	34					I34V					
	321					I312V					
	386					N386K					
	432					K432E					
	13						V13I				
	314						I314M				Antigenic sites
	188							I188T			
	77								G77R/K		
	81								V81A		
	449								N449D		

**FIGURE 4:**
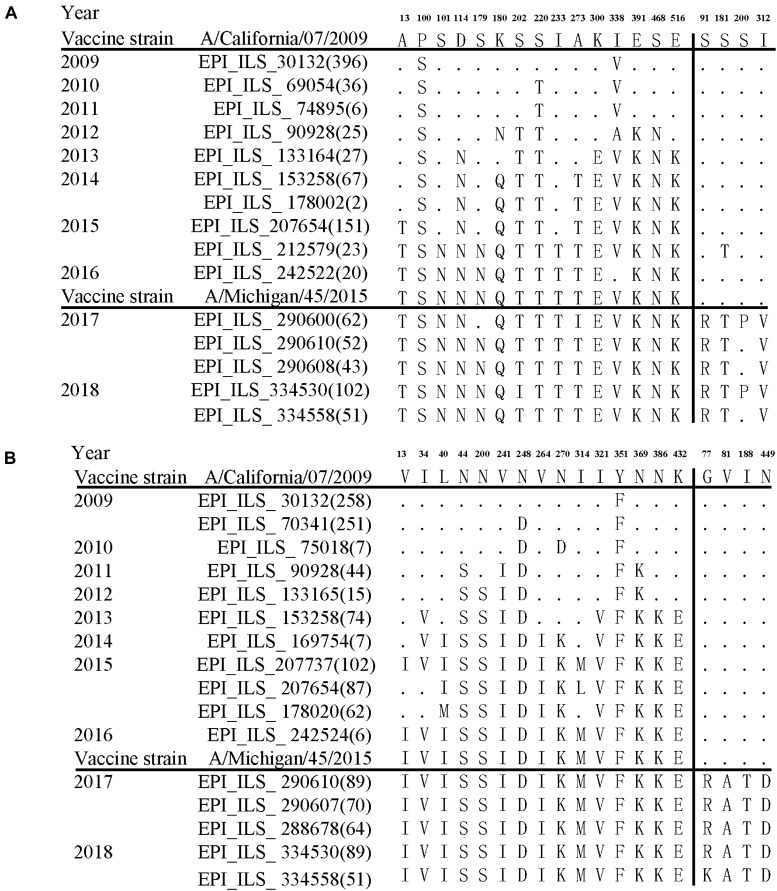
Amino acid variations of HA **(A)** and NA **(B)** glycoproteins of A/H1N1pdm virus during 2009–19 compared to the vaccine strains. The small dots indicate identical amino acids to the vaccine stain A/California/07/2009. The number of identical sequences is shown in parenthesis.

Further comparison with two WHO-recommended vaccine strains showed that the fixed mutations covered almost all the main difference between the circulating A/H1N1pdm lineages and the vaccine strains (Figure 4). Compared to the 2009 vaccine strain, majority of the mutations in HA and NA genes of circulating lineages were gradually accumulated during 2011–2016. Interestingly, all fixed mutations (S91R, S181T, S200P and I312V in HA and G77R, V81A, I188T, and N449D in NA) causing difference to the 2015 vaccine strain appeared in or after 2017 (Figure 4), which may enable the newly emerging lineage in 2017 to escape the immunity induced by the new vaccine.

### Population Dynamics of the A/H1N1pdm in China

**FIGURE 5:**
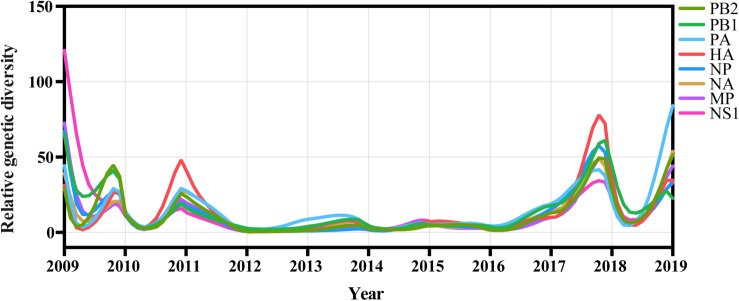
Population dynamics of genetic diversity of A/H1N1pdm virus in China during 2009–2019. The relative genetic diversity of each gene was estimated using the Gaussian Markov Random Fields (GMRF) Skyride model.

**FIGURE 6:**
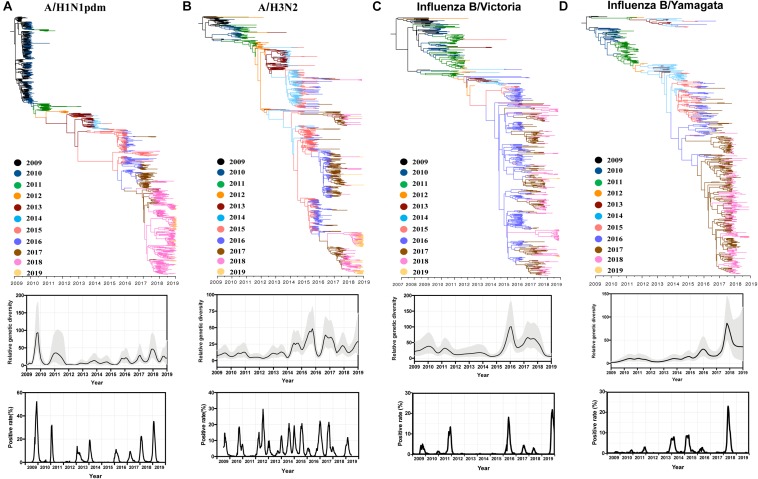
Comparative phylogenetic analysis, population dynamics and positive rate of A/H1N1pdm **(A)**, A/H3N2 **(B)**, influenza B/Victoria **(C),** and influenza B/Yamagata **(D)** viruses. Phylogenies were inferred using the strict clock model and relative genetic diversity estimated using the Gaussian Markov Random Field (GMRF) model. The branches were colored by sampling year. Solid black lines in the GMRF plot represent mean relative genetic diversity, and the gray shades indicate the 95% HPD (highest probability density) intervals. The positive rate of A/H1N1pdm virus (2009–2019), A/H3N2 (2009–2019), influenza B/Victoria (2015–2019), influenza B/Yamagata (2015–2019) by week of specimen collection in China was retrieved from Chinese National Influenza Center.

We inferred the relative genetic diversities of all eight available genomic segments of A/H1N1pdm virus using the GMRF skyride coalescent model. A strong seasonal fluctuation was observed in the relative genetic diversity of A/H1N1pdm virus from 2009 to 2019 with all eight segments showing consistent oscillating patterns (Figure 5). These results indicate that the A/H1N1pdm virus rapidly adapted to human host and evolved into seasonal influenza under strong bottlenecks between seasons after introduction into human population. Interestingly, higher genetic diversity with greater frequency of oscillation was observed during two periods of 2009–2011, and 2017–2019; in contrast, lower genetic diversity with relatively weak seasonal fluctuation maintained from 2012 to 2016 (Figure 5). The seasonal fluctuation pattern of relative genetic diversity seemed to be less likely affected by the sampling bias (i.e., greater sampling) in some years (Supplementary Figure S11), and was consistent with the epidemic pattern (Figure 6A). In particular, the elevated genetic diversity during 2017–2019 reflected an increased activity of the A/H1N1pdm virus in the population during this period (Figure 6A).

We further analyzed all available HA sequences of A/H1N1pdm, A/H3N2, and influenza B Victoria and Yamagata lineages, and compared their relative genetic diversities (Figure 6). A similar genetic diversity pattern of A/H1N1pdm virus was observed even if more HA sequences were used (Figures 5, 6A). Distinct from A/H1N1pdm virus, the A/H3N2 virus exhibited a weak seasonal fluctuation of genetic diversity during 2009–2014, followed by a strong seasonal fluctuation during 2014–2019 (Figure 6B). Two influenza B lineages, Victoria and Yamagata had similar genetic diversity patterns with almost invariant relative genetic diversity from 2009 to 2015 (especially for Yamagata lineage) followed by elevated genetic diversities with strong seasonal fluctuation (Figures 6C,D). A common feature of the four influenza virus lineages was that their relative genetic diversities were elevated since 2016, which might be associated with a sharp increase in influenza cases in China since the winter-spring season of 2017–2018.

### Spatial Dynamics of the A/H1N1pdm in China

**FIGURE 7:**
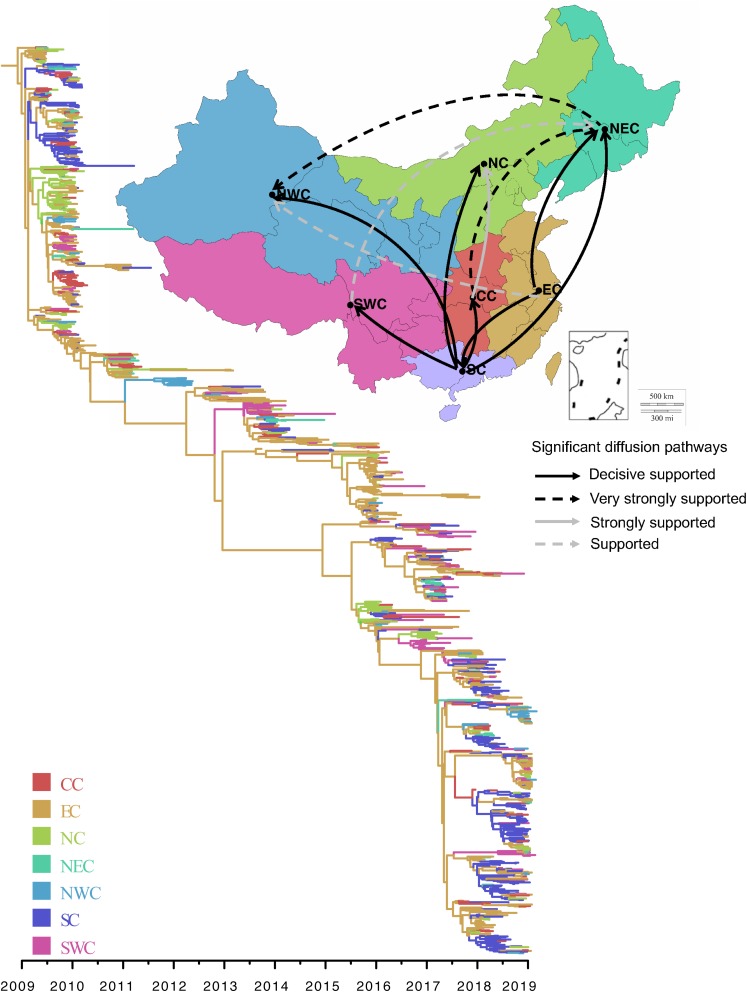
Spatial diffusion of A/H1N1pdm virus. Thickness of lines represents supported migration rates: solid black arrows, decisive support with BF > 1000; dashed black arrows, very strongly supported with 100 < BF < 1000; solid gray arrows, strongly support with 10 < BF < 100; dashed gray arrows, support with 3 < BF < 10. In the phylogenetic tree, colored branches represent different regions. North China (NC), Northeast China (NEC), Northwest China (NWC), Central China (CC), East China (EC), South China (SC), and Southwest China (SWC).

We performed phylogeographic analysis to assess the spatial transmission pattern of A/H1N1pdm virus in seven geographical regions of China (North China, Northeast China, Northwest China, Central China, East China, South China, and Southwest China). The phylogenetic analysis showed that all A/H1N1pdm strains were rooted with the strains from East China, and the main trunk of the MCC tree was also characterized by an origin of East China (Figure 7 right panel). As a whole, two major epicenters were observed in East China and South China (Supplementary File S2: video), both of which mediated seven significant migration links with mean rates of 0.517–2.148 (decisive support with BF > 1000) (Figure 7 left panel and Supplementary Table S3). Two of the migration routes were from East China to South China and Northeast China. Other five migration routes were involved in South China, from where the virus was dispersed to Central China, Southwest China, North China, Northeast China, and Northwest China. These results indicate that East China and South China, as the primary seeding populations, played important roles in national migration of A/H1N1pdm virus. Very strongly supported migration links were also observed from Central China to Northeast China, and from Northeast China to Northwest China.

Further spatiotemporal migration analysis showed that the A/H1N1pdm virus spread from East China to South China and North China in about 2010, from where the virus further spread to other geographical regions in 2011 (Supplementary File S2: video). During 2012–2016, there were rare migration events to occur possibly due to low activity of the virus. Since 2017, East China, South China, and North China formed three new epicenters of the A/H1N1pdm pandemic, and spread the virus to other geographical regions.

To see if the migration pattern from East and/or South China to other parts of China still existed when the sequences from outside of China was added, further Bayesian phylogenetic analysis was performed using 825 A/H1N1pdm HA sequences, including 428 sequences from six geographical regions of the world and 397 sequences from China. The sequences from outside of China were randomly and evenly selected from North America, South America, Europe, South-east Asia, Africa, and Australia. Similarly, the sequences in China were also randomly and evenly selected from seven main regions of China. Except for East and South China, other parts of China also had imported viral strains from outside of China (Supplementary File S3: video). Importantly, the main domestic migration patterns were still observed from East- and/or South China to other parts of China.

## Discussion

The main force driving the evolution of influenza viruses is the adaptation to the new hosts and/or the escape from pre-existing host immunity (Petrova and Russell, 2018; Han et al., 2019). Nucleotide substitutions (amino acid mutations) and reassortment are two major patterns involving the evolution of influenza viruses and can cause antigenic drift and antigenic shift, respectively (Taubenberger and Kash, 2010). Since its origin via triple reassortment and introduction into human population in 2009, A/H1N1pdm virus rapidly spread among human, and replaced the previous seasonal A/H1N1 virus to be the season’s dominant strain. Intra-subtype reassortment and adaptive amino acid substitution are believed to be the main driver for the evolution of A/H1N1pdm virus in humans (Holmes et al., 2005; Westgeest et al., 2014; Petrova and Russell, 2018; Virk et al., 2019; Wille and Holmes, 2019). A transition of selection pressure from host adaptation in pandemic phase (2009–2010) to immunological escape in the post-pandemic period (after 2011) was previously observed in A/H1N1pdm virus (Su et al., 2015). In this study, we performed a comprehensive investigation of the evolutionary dynamics of A/H1N1pdm virus from 2009 to 2019 in China, which provides important insights into the epidemiology, transmission and prevention of the virus.

We found a trend of decrease from 2009 to 2017, followed by increase since 2017 in the dN/dS value of A/H1N1pdm virus. As previously observed, higher dN/dS ratio during the 2009–2010 pandemic season might be a consequence of relaxed selection constraint after entering the new host population, and be associated with its rapid population expansion among human population (Su et al., 2015). The decrease of dN/dS value from 2011 to 2017 might be associated with selective constraints to maintain the functional fitness (e.g., receptor binding) of the virus. The increase in dN/dS values during the 2018–2019 pandemic season might be a consequence of recent virus expansion and increased selection pressure from pre-existing host immunity induced by prior infections or vaccination. The annual variation of relative genetic diversity well matches the seasonal epidemics of this virus. A trend of decrease from 2009 to 2017 followed by increase since 2017 was observed in the relative genetic diversity of the A/H1N1pdm virus. Higher relative genetic diversity during the 2009–2010 matched early rapid population expansion of A/H1N1pdm virus among human population. Decreased relative genetic diversity might be a consequence of transmission bottlenecks (Petrova and Russell, 2018). Apart from A/H1N1pdm, A/H3N2 and two lineages of influenza B virus are also the major agents responsible for the seasonal influenza pandemics. The four influenza subtypes are often co-circulating and compete with each other, resulting in the epidemic dominance of one or two subtypes. We found that all four viruses had consistent, elevated relative genetic diversities during 2018–2019. However, it is unclear why and which factors determine the high level of activity of all four influenza subtypes during 2018–2019.

HA and NA are the most crucial proteins for infection and transmission of influenza viruses. HA is responsible for cell attachment by binding to sialic acid receptors on host cell surface and entry by mediating membrane fusion (Taubenberger and Kash, 2010). The HA gene consists of two subunits, HA1 (327 amino acid residues) and HA2 (222 amino acid residues) (Caton et al., 1982). The HA1 subunit forms a globular head of the HA, including the receptor binding site and five antigenic sites (Sa, Sb, Ca1, Ca2, and Cb) (Gerhard et al., 1981; Igarashi et al., 2010), and is the primary target for human adaptive immune response to influenza viruses. NA is responsible for the release of new virions from infected cells by cleaving the bonds between HA and sialic acid (Cohen et al., 2013). From 2009 to 2019, continuous genetic changes were observed in HA and NA glycoproteins of A/H1N1pdm virus, and 15 amino acid changes were fixed in each of both proteins along the main trunk of the phylogenetic trees. Among these fixed mutations in HA, majority (80%) occurred on the HA head and six (S202T, K180Q, S179N, S181T, S91R, S200P) were situated either at antigenic sites or in proximity to the receptor-binding pocket. For NA, two fixed mutations were found to be situated at antigenic sites (Gao et al., 2019; Yasuhara et al., 2019). Importantly, we found that new genetic variants of A/H1N1pdm virus appeared almost every 1–2 years and replaced previously existing antigenic variants to dominate the forthcoming influenza pandemic. The frequency of emergence of new A/H1N1pdm antigenic variants appeared to be higher than previously thought (Chen and Holmes, 2008; Bedford et al., 2015; Vijaykrishna et al., 2015).

Influenza vaccination is the primary measure to prevent and control seasonal influenza virus infections (Wang et al., 2018). Recent study showed that individual immune selection pressure has weak effects on the evolution of seasonal influenza viruses (Han et al., 2019). However, the effect of vaccination on influenza evolution is less clear. The vaccine-induced effect might be underestimated because of the low influenza vaccine coverage rate (<10% of the world population) (Partridge and Kieny, 2013). The first A/H1N1pdm vaccine was developed based on A/California/07/2009, and was licensed in 2009. It was recommended to be used globally by WHO until to 2017 when a new A/Michigan/45/2015-based vaccine was licensed to keep pace with the emergence of new circulating variants (Petrova and Russell, 2018). From 2009 to 2016, newly emerging A/H1N1pdm strains gradually accumulated 15 amino acid changes in each of both HA and NA glycoproteins compared to the A/California/07/2009 vaccine strain. The accumulation of these mutations might be caused by adaptive immunity induced by prior infections with old antigenic variants and vaccination during this long-term period. Compared to the A/Michigan/45/2015 vaccine strain, the newly emerging A/H1N1pdm lineages in 2017 rapidly evolved 3–4 amino acid changes in each of HA and NA glycoproteins during a short-term period, and two amino acid changes (S91R and S181T) on HA are associated with the Sa and Cb antigenic sites. Relate to high vaccination coverage (35.5–70.4%) in developed countries (e.g., the United States and Europe), China has a very low influenza vaccination coverage rate (about 1.5–2%). Therefore, the accumulation of these amino acid changes in the 2017 A/H1N1pdm lineages may be less likely related to the use of the vaccine (at least in China), but were more likely generated under the selective pressure of pre-existing immunity induced by the predominant A/H1N1pdm strains. Because this study focuses on the phylodynamics of A/H1N1pdm virus, hemagglutination inhibition (HI) assay was not performed to measure the effects of fixed amino acid changes on the antigenicity of the virus. Furthermore, we also did not conduct experiments to validate functional effects of the changes in NA glycoprotein. Bayesian phylogenetic analyses suggested multiple introduction of A/H1N1pdm virus into China from other regions of world. The substitutions (HA: 91, 181, 202, and 312; NA: 77, 81, 188, and 449) occurring in the newly emerging A/H1N1pdm variants in 2017 were also observed in the strains circulating globally during 2017–2019, but not in the strains before 2017 (data not shown). These amino acid mutations might lead to reduced efficacy of the WHO-recommended A/Michigan/45/2015 vaccine due to high sequence similarity to the predominant circulating strains. Wide spread of the 2017 newly emerging A/H1N1pdm lineages in population implies an urgent need to update the A/Michigan/45/2015-based vaccine, and as a response, WHO updated the vaccine composition using A/Brisbane/02/2018 strain for 2019–2020.

Population density and humidity are demonstrated to be the main factors affecting the seasonal epidemics of influenza viruses (Lowen et al., 2007; Shaman and Kohn, 2009; Tamerius et al., 2013). China is a densely populated country, covering complex temperature zones. East China and South China not only are the most important hubs for domestic and international travel, but also densely populated areas. It is not surprising to find that the earliest A/H1N1pdm strains in China originated from East China. Furthermore, East China and South China were found to be two major epicenters responsible for the national migration of the A/H1N1pdm in several epidemic seasons. Suitable climatic condition for influenza virus transmission, high population density, and increasing frequency of domestic and international population mobility via air travel or high-speed rail could explain the seeding hierarchy of East China and South China in influenza epidemics in China. Therefore, to strengthen influenza surveillance in East China and South China will aid the early finding of newly emerging variants and forecast their epidemics in other regions. On the other hand, although frequent introductions of A/H1N1pdm virus lineages into China from outside were observed, they did not change the main national migration patterns of A/H1N1pdm from East China and/or South China to other parts of China. These results suggest that A/H1N1pdm might be persistently maintained in China since its initial introduction in 2009. Subsequent multiple introductions of the virus from outside to China might contribute to its genetic diversity, but have less influence on the domestic pandemic and migration.

In summary, our study provides a comprehensive description on the spatiotemporally evolutionary dynamic of A/H1N1pdm virus in China during 2009–19 and identifies a series of amino acid mutations to be fixed in HA and NA glycoproteins. We found that elevated dN/dS value and relative genetic diversity of A/H1N1pdm virus occurred during 2018–2019, and inferred that simultaneous activities of A/H1N1pdm virus with A/H3N2 and two lineages of influenza B virus during this period were associated with the severe seasonal influenza pandemics. Furthermore, East China and South China were found to play a crucial seeding role in the spread of A/H1N1pdm virus, which is of major significance for influenza prevention and control in China.

## Data Availability Statement

The datasets generated for this study can be found in the GISAID database (http://platform.gisaid.org/, accession numbers of EPI1494609 to EPI1495079 and EPI1497986 to EPI1498327).

## Ethics Statement

The collection of samples was approved by the Ethics Committees of Shanghai Children’s Medical Center. Oral or written informed consents were obtained from children’s parents or guardians before sample collection.

## Author Contributions

CZ and YM contributed to the study design and interpreted the results. KL assembled the whole genome sequences. YY collected the clinical samples. YM, YY, and JQ performed the experiments. YM performed the phylogenetic analyses. KL and Y-HZ contributed to the methodology. JY analyzed the data of the influenza positive rates. CZ, YM, JQ, and SL interpreted the results. CZ and YM wrote the manuscript. LP provided critical suggestions on the results and contributed to revision of the manuscript. All authors read the manuscript and approved the submitted version.

## Conflict of Interest

The authors declare that the research was conducted in the absence of any commercial or financial relationships that could be construed as a potential conflict of interest.

## Footnotes

1 http://platform.gisaid.org/

2 http://datamonkey.org
