# Supplementary material for: The Phylodynamics of Seasonal Influenza A/H1N1pdm Virus in China Between 2009 and 2019
Source: Front Microbiol. 2020 Apr 28;11:735. doi: 10.3389/fmicb.2020.00735 (PMC7228120; doi:10.3389/fmicb.2020.00735)
Supplement: Supplementary file 4 [file Data_Sheet_4.docx]

Supplementary Material

**Supplementary file 1. Viruses sequenced in this study.**

**Supplementary file 2. (video) Animated version of the data shown in Figure 7.** Black lines indicate all viral movement pathways between seven major China regions. The size of the red circle represents the number of outbreaks in targeted area.

**Supplementary file 3. (video) Migration of A/H1N1pdm virus in global (EU, AF, SEA, AUS, NAM, and SAM) and China (SC, SWC, CC, EC, NWC, NC, and NEC).** Black lines indicate all viral movement pathways between seven major China regions. The size of the red circle represents the number of outbreaks in targeted area.

**Supplementary Table 1. Adaptive evolution analysis of HA gene of A/H1N1pdm virus in China from 2009 to 2019.**

| years | dN/dS* | Number of positively selected sites by M8+BEB (amino-acid position) | Number of positively selected sites by MEME (amino-acid position) |
| --- | --- | --- | --- |
| 2009-2010 | 0.45 | 12 (14,142,148,152,187,210,217,234,282,384,460) | 2 (145,239) |
| 2010-2011 | 0.31 | 5 (155,180,190,238,389) | 1 (391) |
| 2011-2012 | 0.30 | 3(180,239,390) | 1 (202) |
| 2012-2013 | 0.33 | 4 (163,221,222,282) | 1 (240) |
| 2013-2014 | 0.29 | 7(1,2,5,178,286,295,538） | 1 (240) |
| 2014-2015 | 0.19 | 4(13,98,166,529) | 4 (169,208,240,451） |
| 2015-2016 | 0.12 | 2(166,204) | 2 (208,240) |
| 2016-2017 | 0.13 | 2(168,180) | 1 (181,240) |
| 2017-2018 | 0.19 | 13(82,127,143,170,179,189,191,257,261,352,401,415,416） | 3 (181,372,433) |
| 2018-2019 | 0.25 | 12(54,145,172,181,191,193,230,263,267,287,373,388) | 5 (2,181,372,433,566) |

* This dN/dS value represents an average over all sites and lineages.

**Supplementary Table 2. Adaptive evolution analysis of NA gene of A/H1N1pdm virus in China from 2009 to 2019.**

| years | dN/dS* | Number of positively selected sites by M8+BEB (amino-acid position) | Number of positively selected sites by MEME (amino-acid position) |
| --- | --- | --- | --- |
| 2009-2010 | 0.28 | 1 (229) | 0 |
| 2010-2011 | 0.26 | 2 (253,332) | 0 |
| 2011-2012 | 0.30 | 3 (256,385,395) | 0 |
| 2012-2013 | 0.29 | 3 (41,266,378) | 0 |
| 2013-2014 | 0.30 | 3 (41,197,446) | 0 |
| 2014-2015 | 0.37 | 4(13,34,313,429) | 0 |
| 2015-2016 | 0.39 | 7 (34,40,95,96,268,312,393) | 0 |
| 2016-2017 | 0.26 | 0 | 1 (41) |
| 2017-2018 | 0.18 | 8 (13,19,23,92,126,198,312,413) | 0 |
| 2018-2019 | 0.20 | 7 (2,73,76,92,142,199,415) | 3 (2,66,389) |

* This dN/dS value represents an average over all sites and lineages.

**Supplementary Table 3. Statistically supported diffusion of epidemiology links of A/H1N1pdm virus between seven geographical regions of China.**

| Migration from | Mean actual rate | Mean indicator | Bayes Factor |
| --- | --- | --- | --- |
| EC to SC | 2.148 | 1 | >1000 |
| EC to NEC | 0.863 | 1 | >1000 |
| SC to CC | 1.052 | 1 | >1000 |
| SC to SWC | 0.617 | 1 | >1000 |
| SC to NC | 0.517 | 1 | >1000 |
| SC to NEC | 0.558 | 1 | >1000 |
| SC to NWC | 0.683 | 1 | >1000 |
| NEC to NWC | 0.948 | 0.995 | 100-1000 |
| CC to NEC | 0.963 | 0.993 | 100-1000 |
| CC to NC | 0.909 | 0.913 | 10-100 |
| EC to NWC | 0.759 | 0.682 | 3-10 |
| SWC to NEC | 0.993 | 0.601 | 3-10 |

EC, East China; SC, South China; NEC, Northeast China; CC, Central China; SWC, Southwest China; NWC, Northwest China; NC, North China.


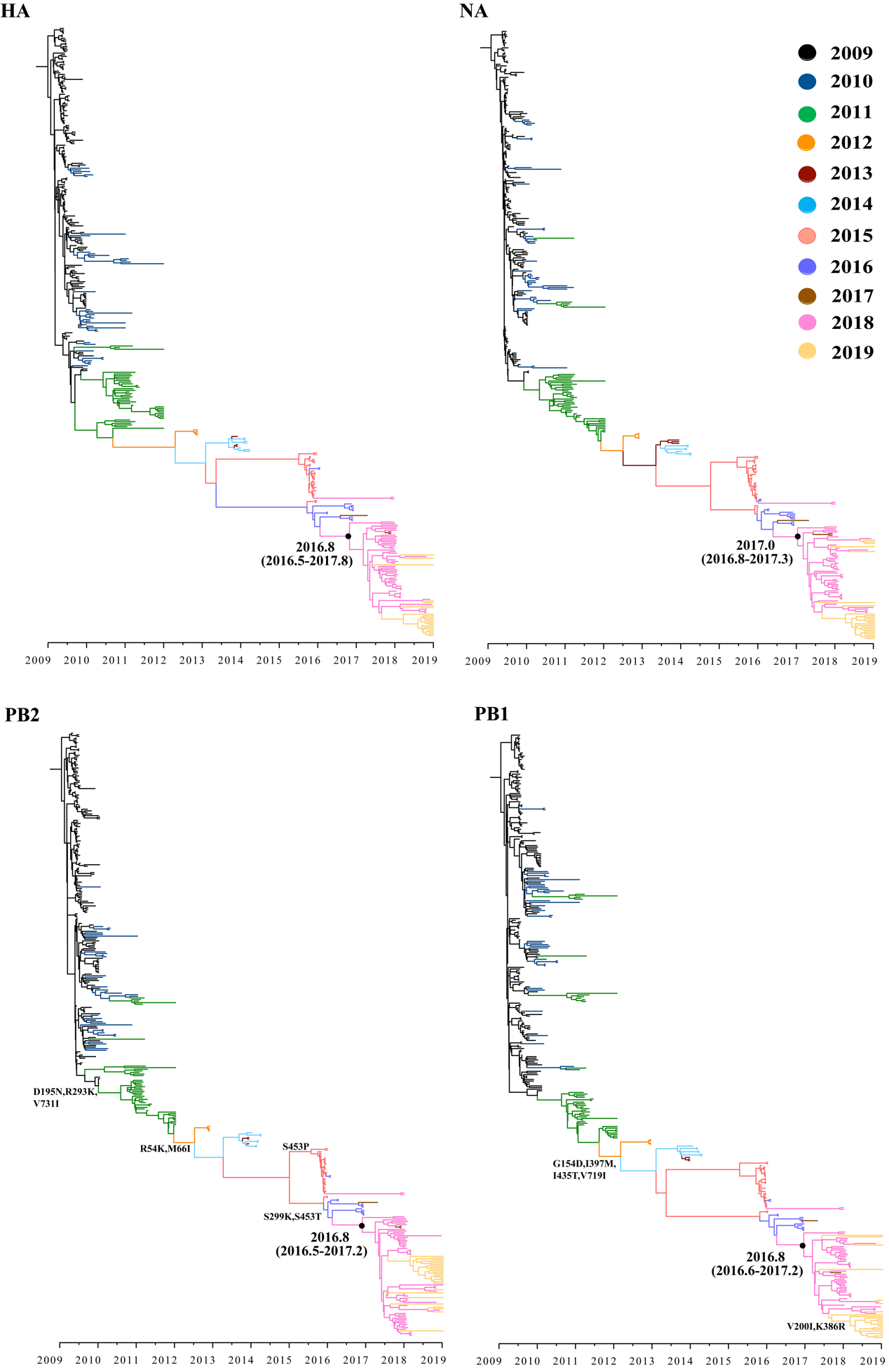


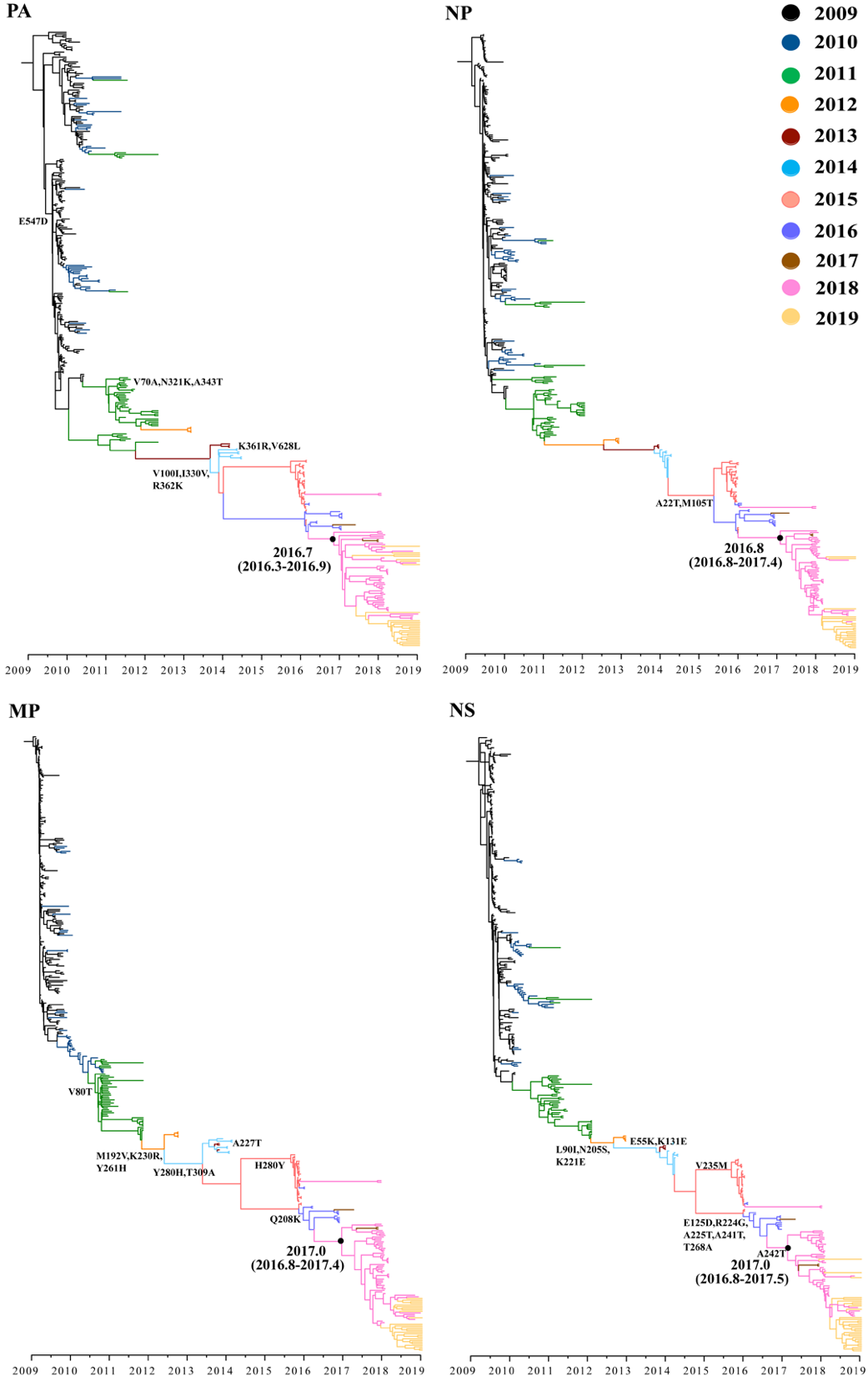


**Supplementary Figure 1. Phylogenies and divergence times of eight genomic segments of A/H1N1pdm virus in China from 2009 to 2019.** Phylogenies were inferred using the strict clock model and Bayesian GMRF skyride coalescent prior. Colored branches represent different year of isolation. Amino acid substitutions are mapped at the major nodes of PB2, PB1, PA, NP, MP, and NS trees.


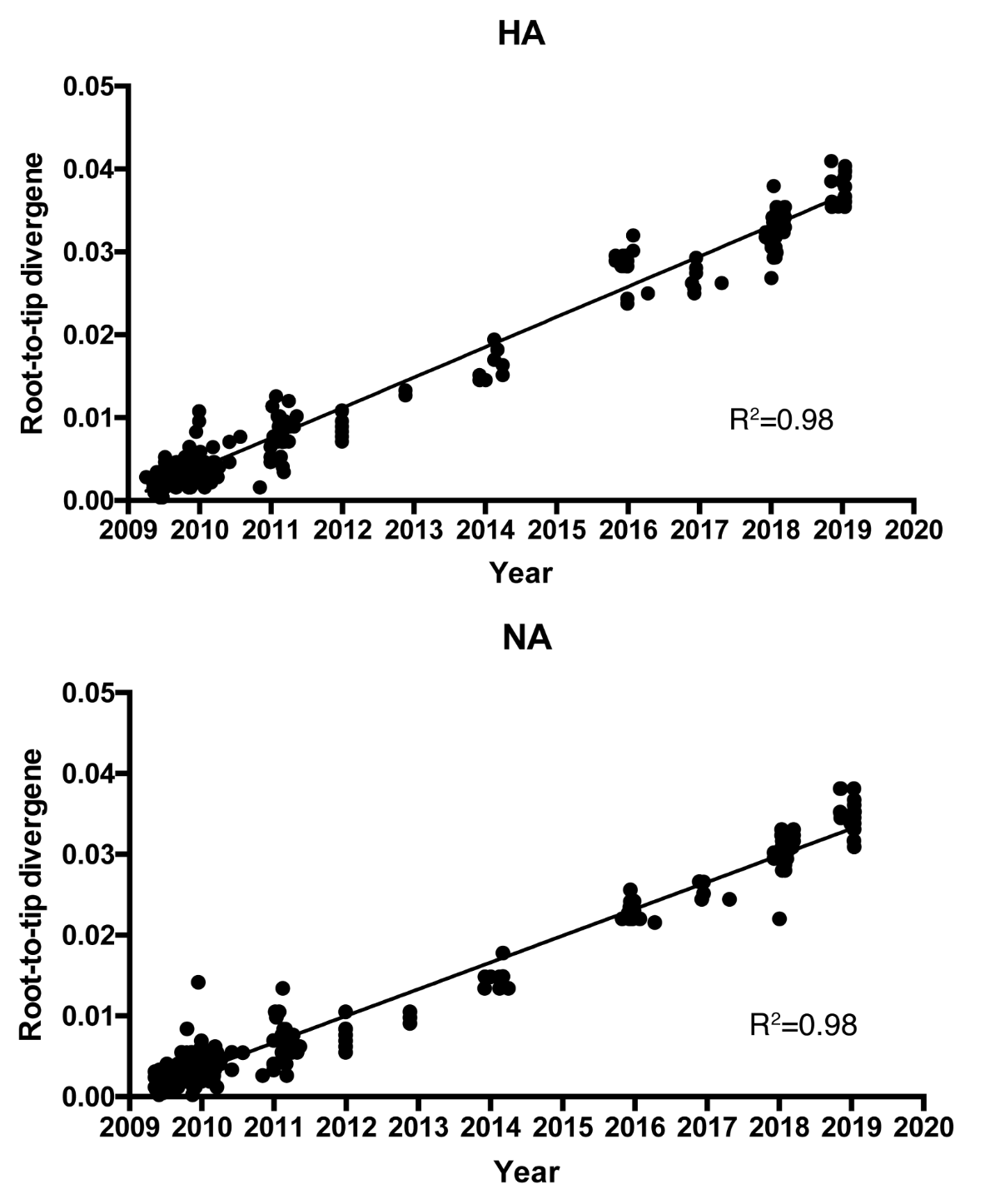


**Supplementary Figure 2. Root-to-tip divergence.** A plot of sampling time versus genetic distance, inferred from trees of HA and NA gene of A/H1N1pdm virus using maximum-likelihood methods. Both HA and NA gene datasets of A/H1N1pdm virus display a strong temporal signal for a molecular clock, as shown by the high R^2^.

**
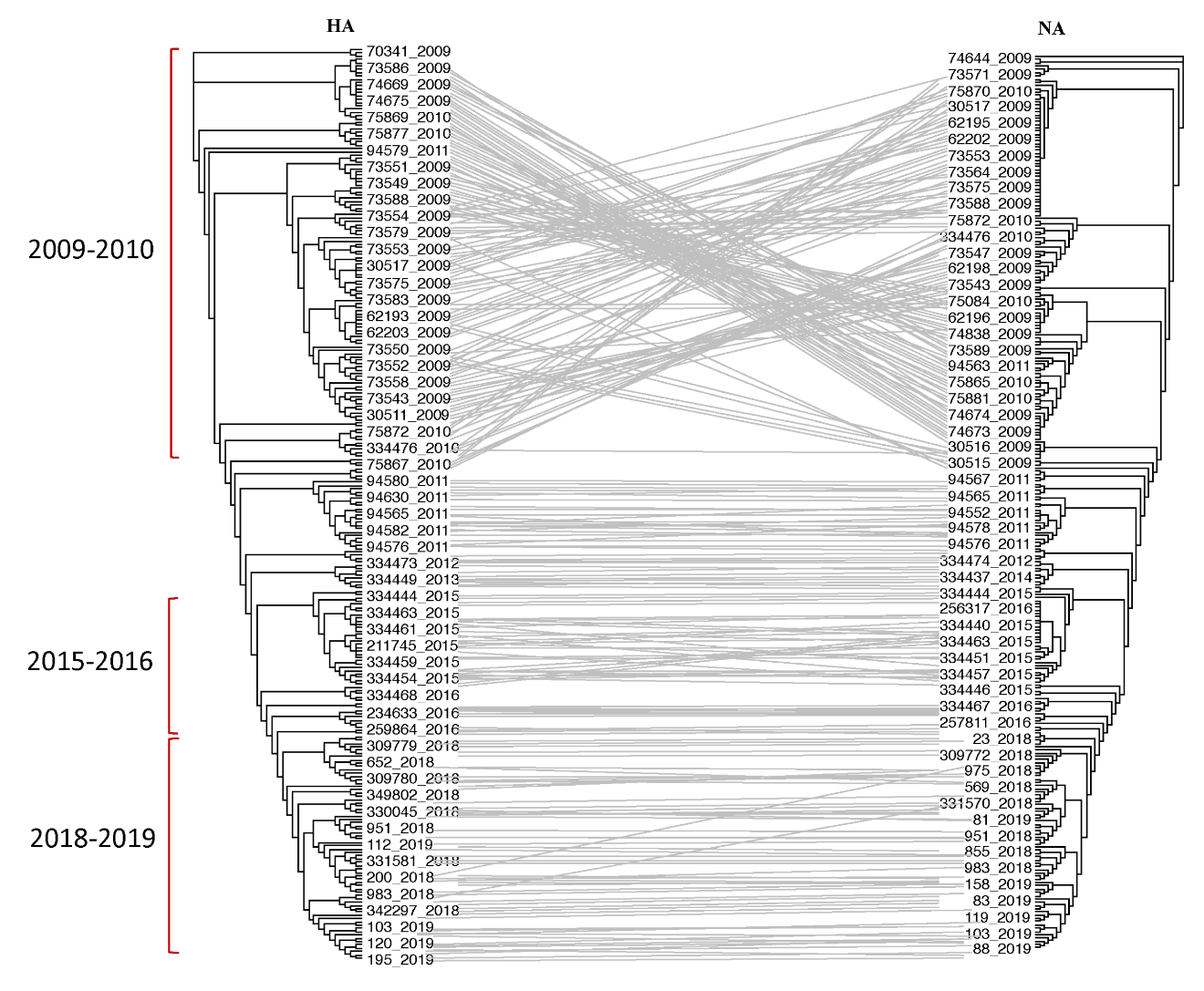
**

**Supplementary Figure 3. Tanglegram of A/H1N1pdm virus HA and NA segments.**

**
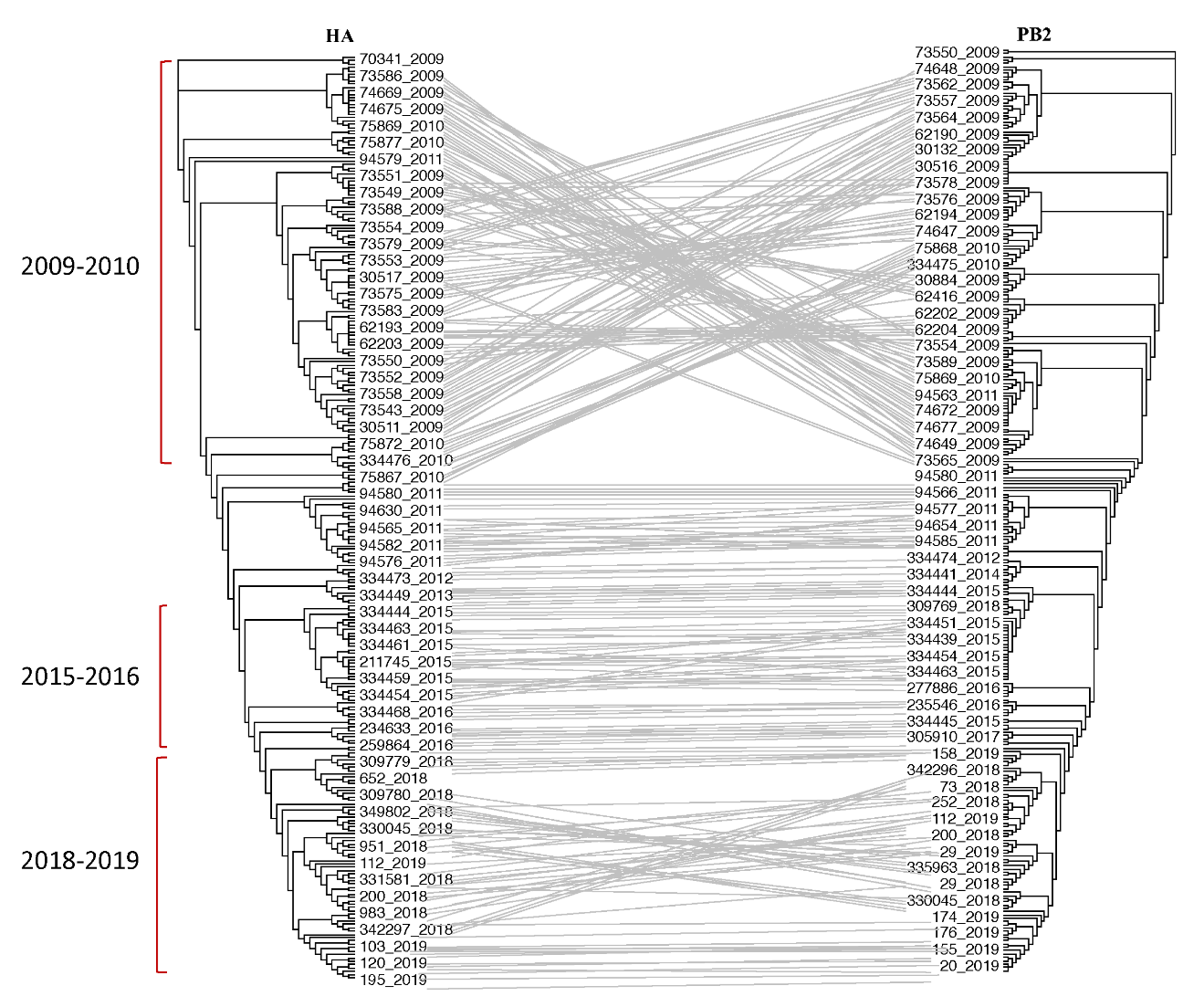
Supplementary Figure 4. Tanglegram of A/H1N1pdm virus HA and PB2 segments.**

**
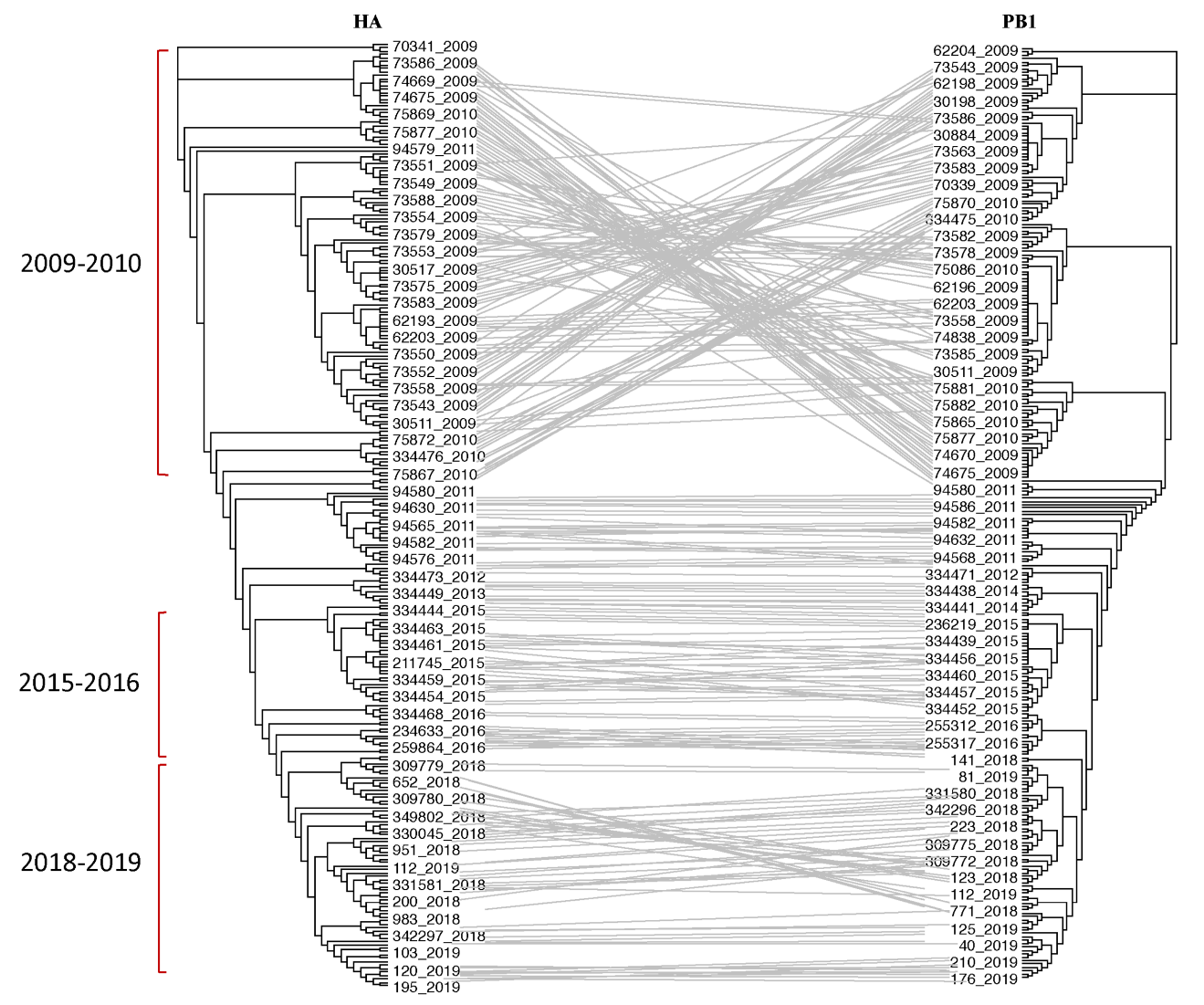
**

**Supplementary Figure 5. Tanglegram of A/H1N1pdm virus HA and PB1 segments.**

**
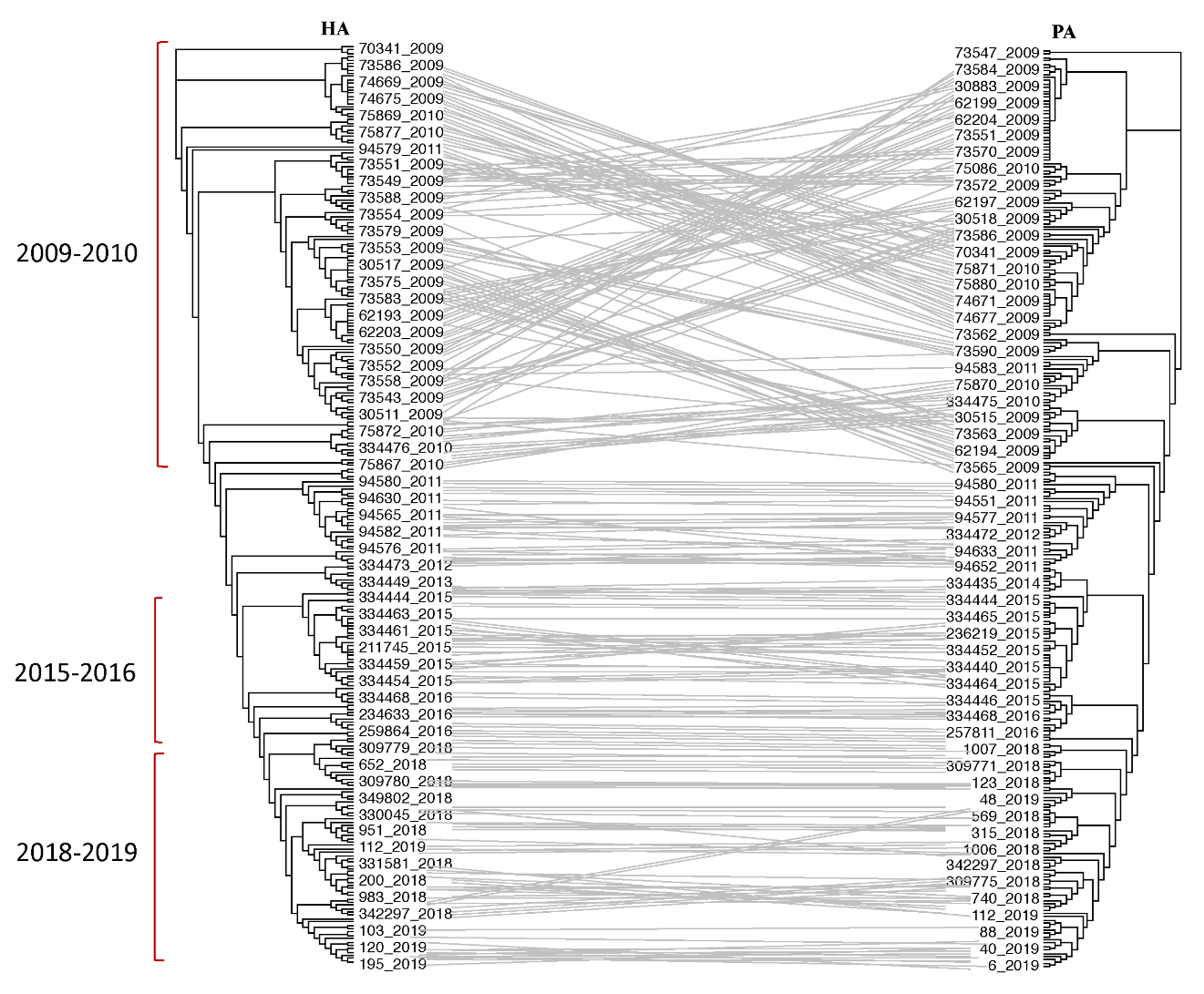
**

**Supplementary Figure 6. Tanglegram of A/H1N1pdm virus HA and PA segments.**

**
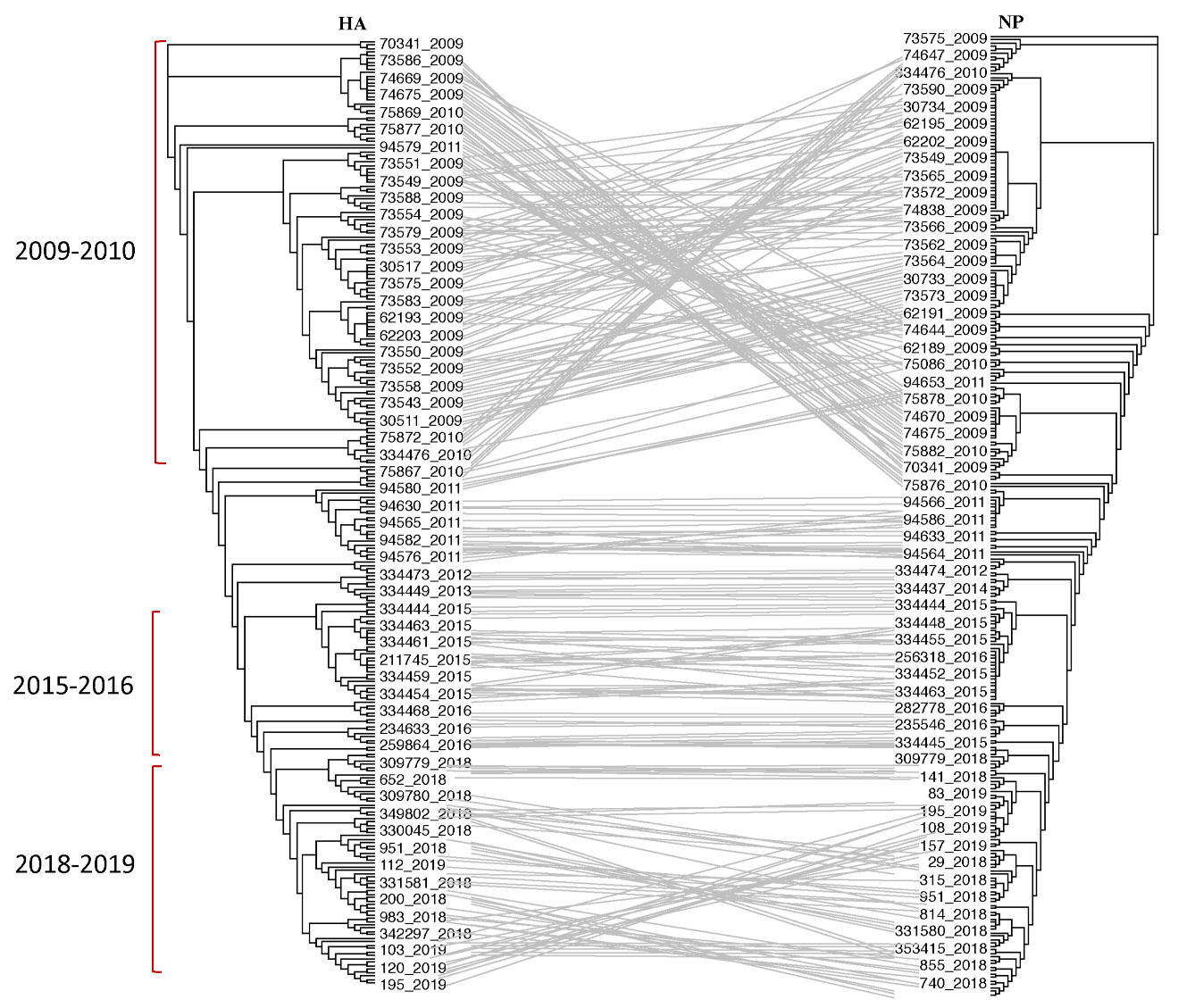
**

**Supplementary Figure 7. Tanglegram of A/H1N1pdm virus HA and NP segments.**

**
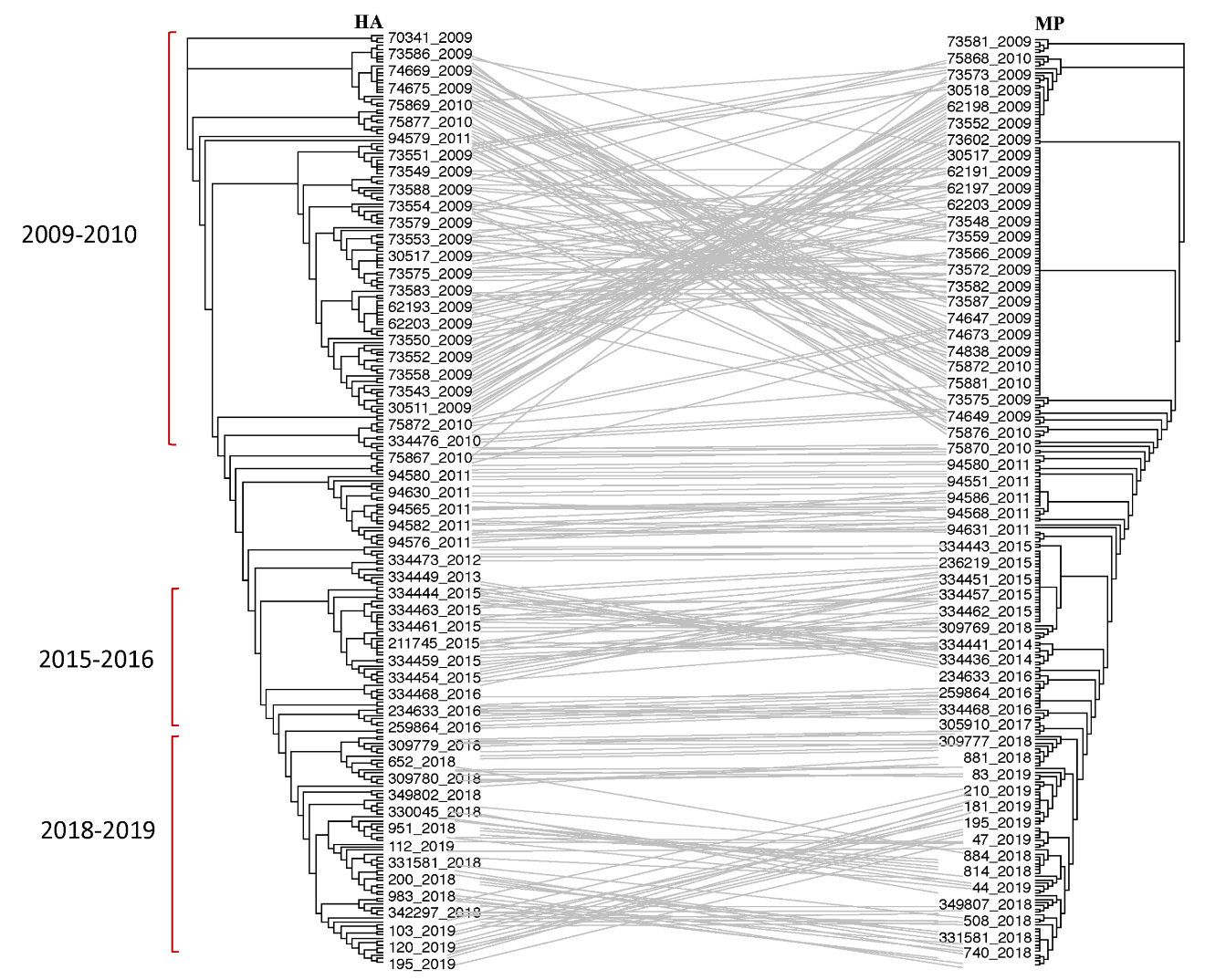
**

**Supplementary Figure 8. Tanglegram of A/H1N1pdm virus HA and MP segments.**

**
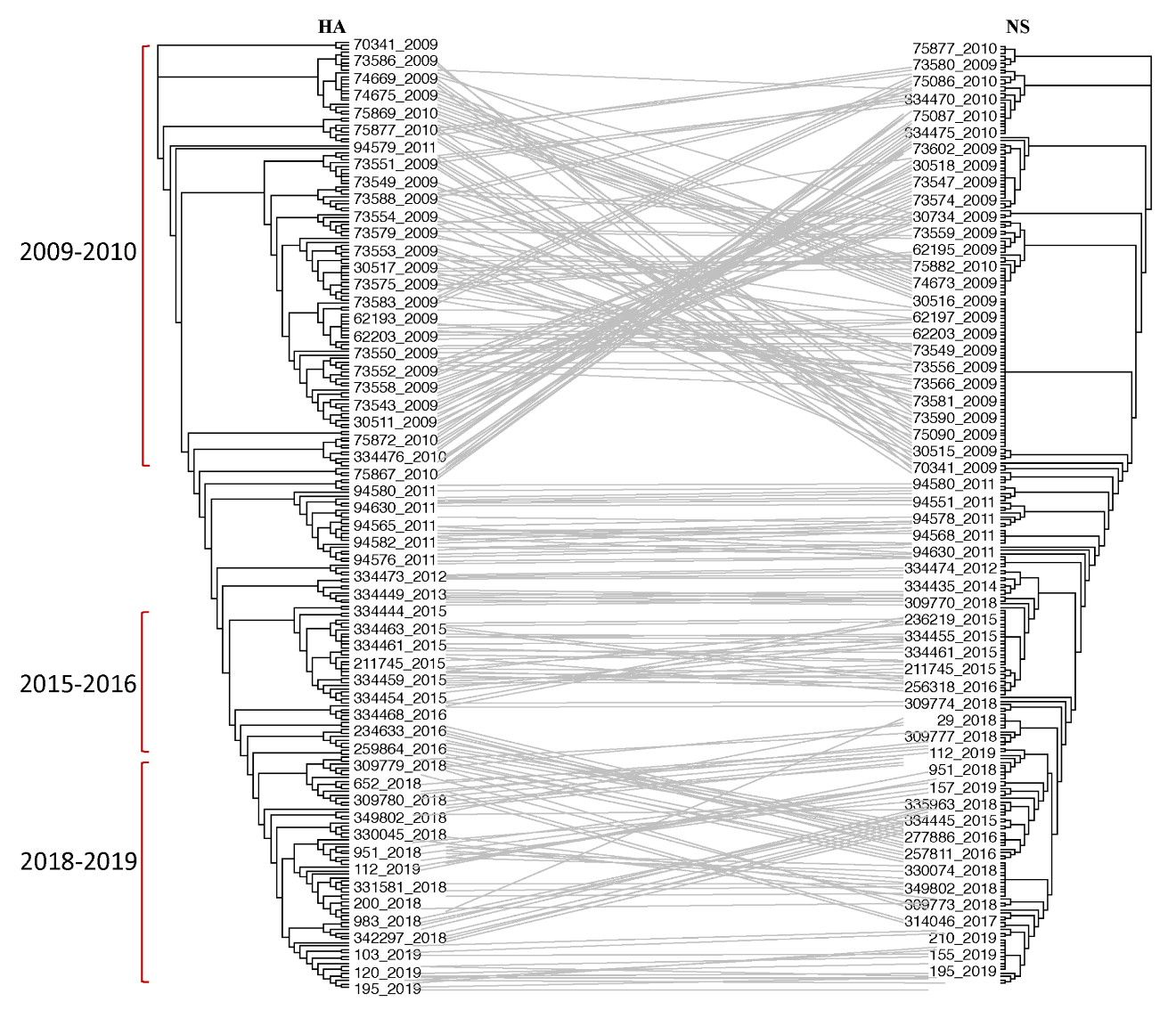
**

**Supplementary Figure 9. Tanglegram of A/H1N1pdm virus HA and NS segments.**

**
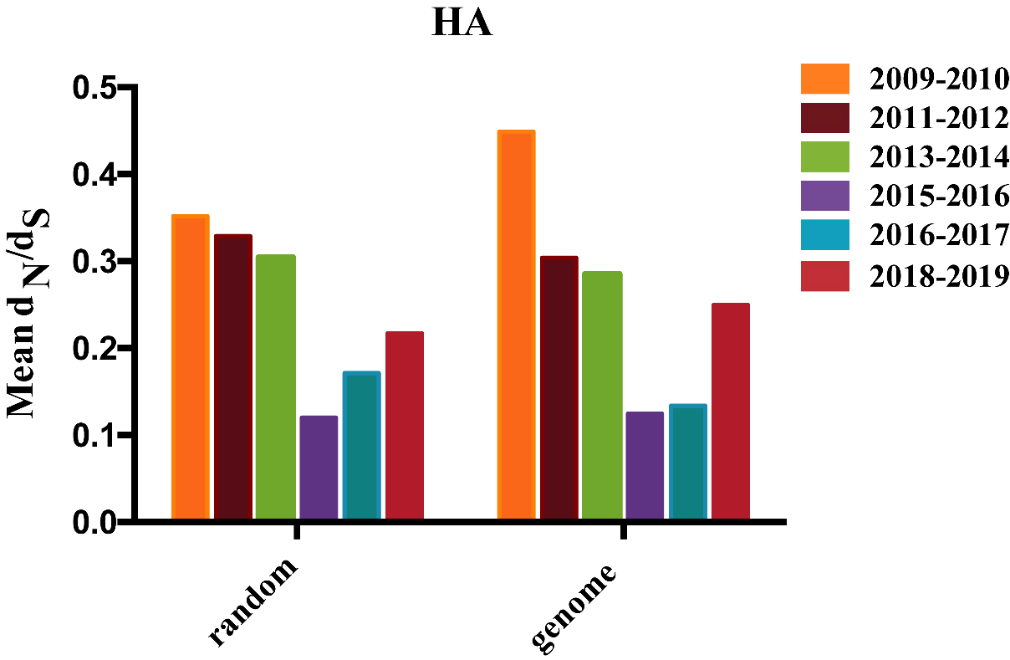
**

**Supplementary Figure 10. Comparison of the mean dN/dS of the HA gene between the datasets from proportionally randomly selected sequences and all available genomic sequences.** Because the genomic sequences were relatively small in some years, to avoid potential sampling bias, we proportionally randomly selected 30 HA gene sequences from each and calculated the dN/dS values of HA gene and compared them with those based on all available genomic sequences. Both datasets generated consistent dN/dS values, suggesting that the dN/dS ratio was less likely affected by different sampling size in different years. The dN/dS value represents an average over all sites and lineages.

**
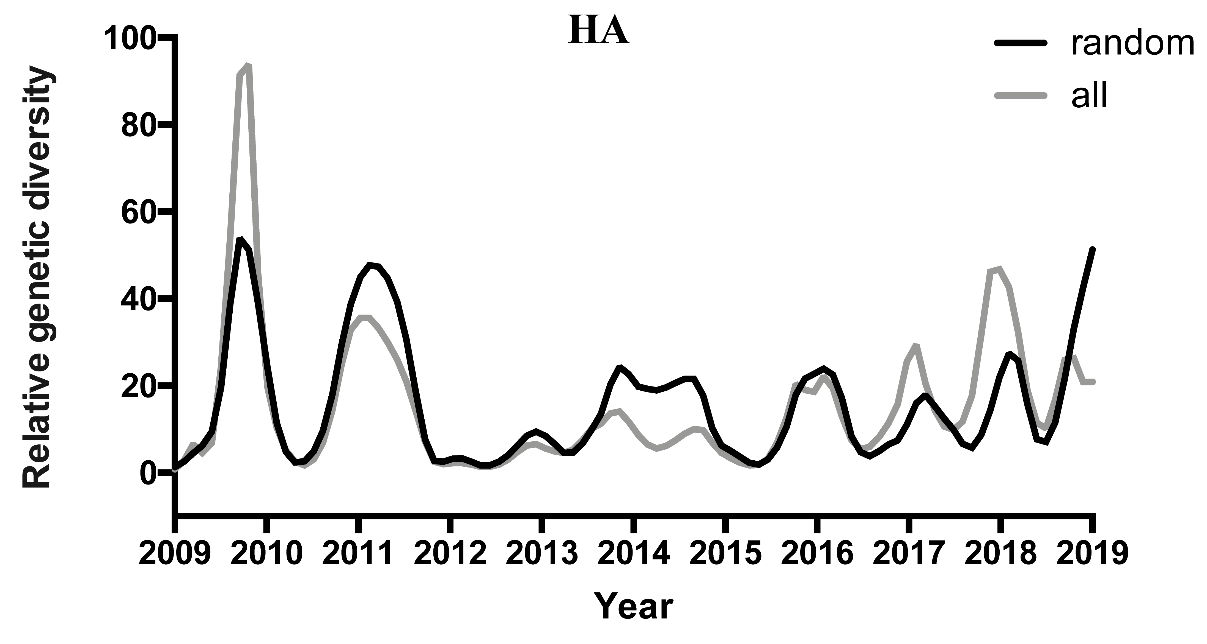
**

**Supplementary Figure 11. Comparison of the genetic diversity of the HA gene between the datasets from proportionally randomly selected sequences and all available genomic sequences.** Because the genomic sequences were relatively small in some years, to avoid potential sampling bias, we proportionally randomly selected 30 HA gene sequences from each and calculated the relative genetic diversity of the HA gene and compared it with that based on all available genomic sequences. Both datasets generated consistent population dynamic trends, suggesting that the result was less likely affected by different sampling size in different years.
